# Anion-Directed Engineering of High-Entropy Layered Double Hydroxides for Oxygen Evolution Catalysis

**DOI:** 10.1021/acsnano.5c15605

**Published:** 2026-02-20

**Authors:** Yin Liu, Xiaorong Jiao, Xingmao Jiang, Congcong Xing, Xueqiang Qi, Xiang Wang, Andreu Cabot

**Affiliations:** † School of Chemical Engineering and Pharmacy, Hubei Key Laboratory of Novel Reactor and Green Chemical Technology, 34756 Wuhan Institute of Technology, Wuhan 430205, China; ‡ State Key Laboratory of Green and Efficient Development of Phosphorus Resources, Wuhan 430205, China; § Catalonia Institute for Energy Research (IREC), Sant Adrià de Besòs, 08930 Barcelona, Catalonia, Spain; ⊥ Institute of Wenzhou, Zhejiang University, 325006 Wenzhou, China; ¶ School of Chemistry and Chemical Engineering, 232838 Chongqing University, Chongqing 400044, China; ∥ ICREA, Pg. Lluis Companys 23, 08010 Barcelona, Spain

**Keywords:** high entropy, layered double hydroxide, electrolysis, anion exchange membrane, oxygen evolution reaction

## Abstract

The development of highly active and durable oxygen evolution reaction (OER) electrocatalysts is crucial for efficient hydrogen production via anion exchange membrane water electrolysis (AEMWE). High-entropy materials have emerged as a versatile platform for electrocatalysis due to their tunable compositions and synergistic effects among multiple elements. Herein, we systematically investigate the influence of different anions, specifically sulfate (SO_4_^2–^), nitrate (NO_3_^–^), and chloride (Cl^–^), on the morphology, surface structure, and OER performance of high-entropy layered double hydroxides (HELDHs) composed of Fe, Co, Ni, Mn, and Zn. Since anions change the microenvironment during the synthesis process, the structures of the prepared nanomaterials vary significantly, from nanosheets and nanowires to porous flower-like structures. These morphological differences directly impact the electrochemically active surface area, oxygen species adsorption, and charge transfer kinetics. Among the evaluated systems, sulfate-based HELDHs (HELDH-SO_4_^2–^) feature a highly porous, interconnected nanosheet architecture and deliver outstanding OER activity and stability in alkaline media, achieving an overpotential of 282 mV at 100 mA cm^–2^ with a low Tafel slope of 45.53 mV dec^–1^. They also demonstrate exceptional performance as anode catalysts in AEMWE. Density functional theory calculations reveal that cation vacancies combined with adsorbed sulfate ions reduce the reaction energy barrier, accounting for the superior activity. These results underscore the pivotal role of anions in tuning the structure–function relationship of high-entropy catalysts and provide a efficient strategy for designing high-performance water oxidation electrocatalysts.

## Graphical abstract

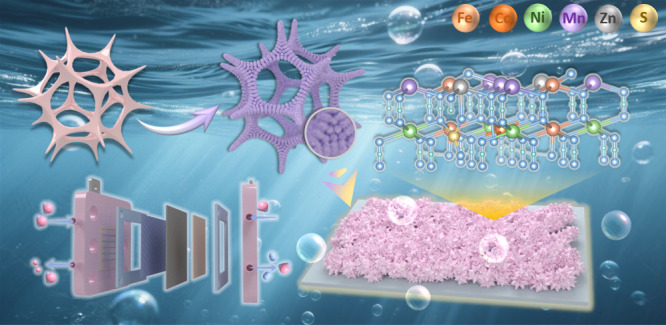

## 1 Introduction

The global transition toward clean and renewable energy has accelerated the development of hydrogen production technologies, particularly water electrolysis.^1,2^ Among the available approaches, anion exchange membrane water electrolysis (AEMWE) is attracting growing interest due to its ability to operate under alkaline conditions, enabling the use of nonprecious metal catalysts and low-cost system components.^3–5^ Despite these advantages, the performance and durability of AEMWE systems are still significantly limited by the sluggish kinetics of the oxygen evolution reaction (OER) at the anode. This challenge underscores the urgent need for efficient, stable, and scalable OER electrocatalysts.^6–8^

Transition metal-based layered double hydroxides (LDHs), especially those based on earth-abundant elements such as Fe, Co, and Ni, have attracted significant interest as efficient and cost-effective OER catalysts in alkaline media.^9–12^ However, the catalytic activity and long-term stability of conventional binary and even ternary LDH systems remain limited, and their low compositional flexibility constrains further optimization of their electronic structure and catalytic properties.^13,14^

To overcome these limitations, the development of high entropy materials (HEMs) has emerged as a powerful strategy to further optimize OER catalyts.^15,16^ By incorporating five or more principal elements in near-equimolar ratios, high-entropy LDHs (HELDHs) can form stable single-phase structures with elevated configurational entropy.^17,18^ This high entropy, along with lattice distortion and a wide variety of tunable active sites, endows HELDHs with exceptional potential for OER catalysis. For example, Wang et al. reported a MnFeCoNiCu HELDH decorated with Au single atoms and oxygen vacancies, which demonstrated both high OER activity and promising long-term stability.^19^ Similarly, Ding et al. synthesized a monolayer HELDH framework that exhibited outstanding OER performance and excellent durability.^20^

While significant efforts have been devoted to optimizing the metal composition of HELDHs, the influence of nonmetallic species, particularly the counteranions of metal salt precursors used during synthesis, on catalyst morphology and surface properties has been largely overlooked. These counteranions can play a crucial role in directing nucleation and growth processes, stabilizing specific crystal facets, and inducing defect formation, all of which profoundly impact the resulting electrocatalytic performance.^21–23^

In a different line of investigation, studies have shown that certain amphoteric metals, such as zinc and aluminum, can undergo selective leaching during electrochemical activation due to their relatively weak metal–oxygen bond strengths and high solubility under alkaline conditions.^24–26^ This leaching process generates abundant cation vacancies and induces local lattice distortions, both of which contribute to enhanced catalytic activity.^27–34^

In this study, we systematically investigate the influence of different anions, specifically sulfate (SO_4_^2–^), nitrate (NO_3_^–^), and chloride (Cl^–^), on the morphology and catalytic activity of HELDHs composed of Fe, Co, Ni, Mn, and Zn. By precisely controlling the anionic environment during synthesis, we uncover the critical role of these anions in modulating the catalyst’s surface structure, electronic configuration, and OER performance. Concurrently, Zn ions undergo selective leaching under alkaline conditions during the electrocatalytic process, leading to the formation of catalytically active cation vacancies. These defects enhance electrical conductivity, increase the exposure of active sites, and facilitate favorable electronic structure modulation. Electrochemical testing demonstrates that HELDH synthesized in the presence of sulfate anions (HELDH-SO_4_^2–^) exhibits outstanding OER performance. Furthermore, this catalyst also delivers excellent activity in AEMWEs, highlighting its strong potential for practical energy applications. Density functional theory (DFT) calculations further elucidate the origin of its superior performance. Overall, this work provides valuable insights into anion-directed morphological engineering and leaching-driven activation strategies, while positioning HELDHs as promising next-generation OER catalysts for sustainable hydrogen production.

## 2 Results and Discussion

### 2.1 Structural Characterization

As detailed in the Experimental Section and illustrated in Figure 1a, a series of HELDH composed of Fe, Co, Ni, Mn, and Zn was synthesized on conductive Ni foam (NF) via a one-step, facile hydrothermal method, using different metal salts to introduce specific ions (HELDH-SO_4_^2–^, HELDH-NO_3_^–^, HELDH-Cl^–^). Scanning electron microscopy (SEM) analyses reveal that the choice of anion plays a critical role in shaping the resulting HELDH morphology. HELDH-SO_4_^2–^ exhibits a porous, ultrathin flower-like nanosheet structure (Figures 1b and S1), whereas HELDH-NO_3_^–^ forms a typical LDH nanosheet array (Figures 1c and S2), and HELDH-Cl^–^ presents a distinct nanowire architecture (Figures 1d and S3).

**Figure 1:**
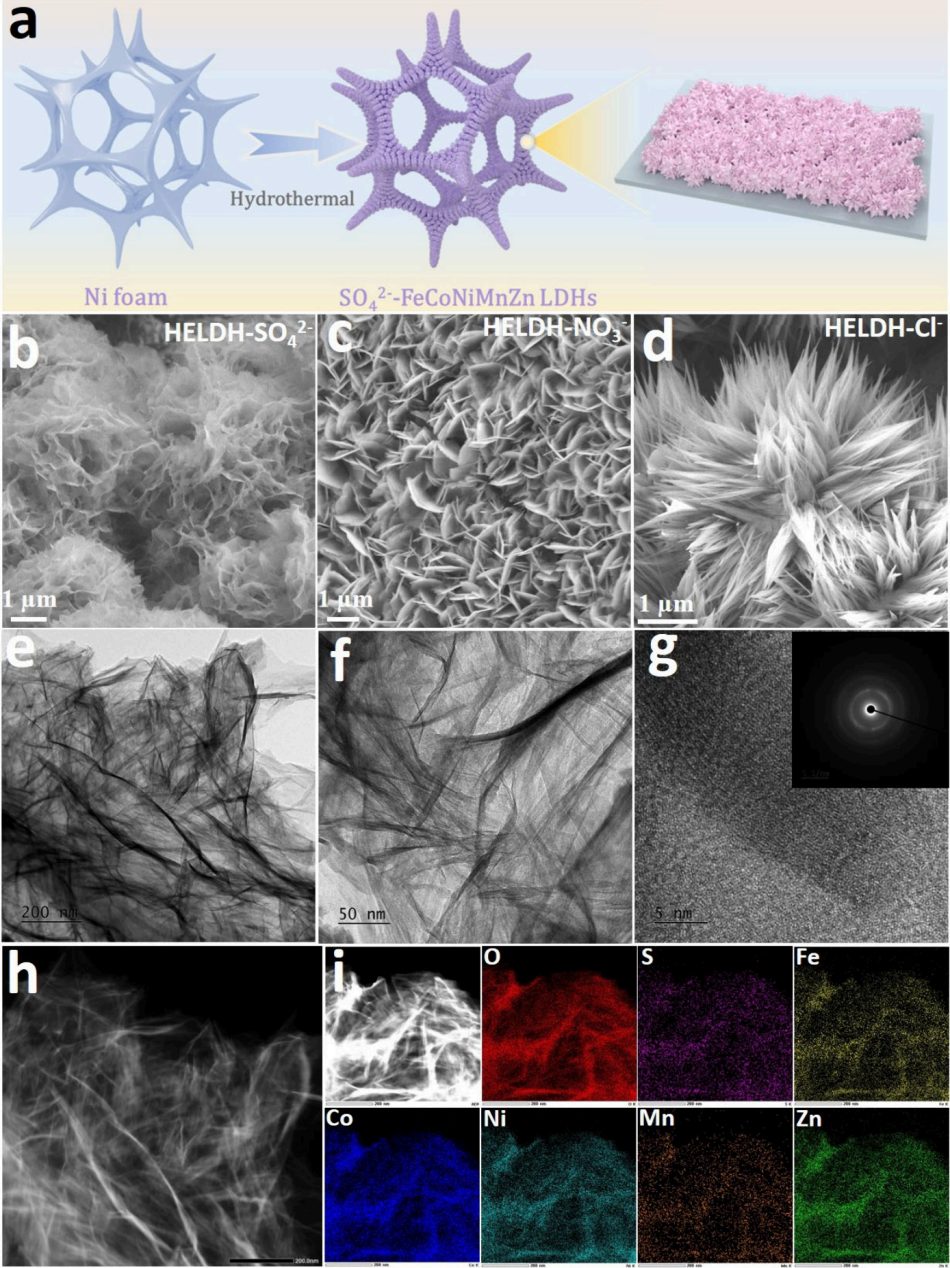
(a) Schematic illustration of the process used to produce HELDH-SO_4_^2–^. (b–d) SEM images of HELDH-SO_4_^2–^, HELDH-NO_3_^–^, and HELDH-Cl^–^, respectively. (e, f) TEM, (g) HRTEM, and (h, i) HAADF-STEM and elemental mapping images of HELDH-SO_4_^2–^. The inset in (g) shows the corresponding SAED pattern.

XRD analysis (Figure S4) reveals that the type of anion used during synthesis has a significant influence on the crystallinity of HELDHs. Among the samples, HELDH-SO_4_^2–^ exhibits notably weaker crystallinity compared to HELDH-NO_3_^–^ and HELDH-Cl^–^, further underscoring the critical role of anion selection in determining the structural properties of HELDHs.

Transmission electron microscopy (TEM) analysis further confirms the ultrathin nanosheet morphology of HELDH-SO_4_^2–^ (Figures 1e-f and S5a-c). Additionally, atomic force microscopy (AFM) imaging (Figure S6) shows that the nanosheets have an average thickness of 1.8 ± 0.2 nm. N_2_ adsorption–desorption curves show HELDH-SO_4_^2–^ exhibits a representative type-IV adsorption isotherm with the largest specific surface area of 187.6 m^2^ g^–1^ and average pore size of 9.6 nm, confirming the presence of mesoporous features. This mesoporous structure provides a high density of exposed surface sites, thereby enhancing electrochemical activity through improved charge transfer and mass transport.^35–38^

High-resolution TEM (HRTEM) and corresponding fast Fourier transform (FFT) analyses (Figure 1g) further confirm the weak crystallinity of HELDH-SO_4_^2–^, in agreement with the XRD results. Interestingly, the lattice fringes observed at the nanosheet edge of HELDH-SO_4_^2–^ (Figure S5d–f) exhibit a spacing of ∼ 6.4 Å, which is markedly larger than the fringe spacing (∼2.4 Å) typically observed for HELDH–NO_3_^–^ and HELDH–Cl^–^. This enlarged periodicity is consistent with an expanded interlayer/basal spacing, most plausibly induced by SO_4_^2–^ intercalation within the HELDH galleries. Supporting this interpretation, FTIR spectra collected after the sulfate removal/exchange treatment (Figure S4b) still display the characteristic sulfate vibrational bands, indicating that SO_4_^2–^ is not merely weakly adsorbed on the external surface. Instead, sulfate remains strongly associated with the hydroxide framework through specific interactions, such as directional hydrogen bonding with layer – OH groups and additional van der Waals/electrostatic stabilization, thereby facilitating interlayer expansion and the formation of the observed superlattice-like structure.

HAADF-STEM imaging and elemental mapping (Figure 1h) demonstrate the uniform distribution of Fe, Co, Ni, Mn, Zn, S, and O throughout the nanosheet structure. TEM, HRTEM, and HAADF-STEM imaging together with elemental mapping for the reference samples HELDH–NO_3_^–^ and HELDH–Cl^–^ are provided in Figures S8–S9. In addition, inductively coupled plasma optical emission spectroscopy (ICP-OES) analysis (Table S1) confirms that the five constituent metals in HELDH-SO_4_^2–^ are present in near-equimolar ratios. Based on these compositions, the calculated configurational mixing entropy (*ΔS*_*mix*_) is 1.6 R, exceeding the commonly used high-entropy threshold of 1.5 R, thereby further supporting the successful formation of a HELDH.

### 2.2 Electrocatalytic Performances

The OER activity of the HELDHs and related binary, ternary, and quaternary LDHs was initially evaluated using linear sweep voltametry (LSV) in a standard three-electrode configuration with 1 M KOH electrolyte. As shown in Figure 2a-b, the HELDH-SO_4_^2–^ exhibited the lowest overpotential (η_100_ = 282 mV, without *iR* compensation) at 100 mA·cm^–2^, outperforming HELDH-NO_3_^–^ (300 mV), HELDH-Cl^–^ (319 mV), and other sulfate-based binary (390 mV), ternary (358 mV), quaternary (323 mV) LDHs. Besides, HELDH-SO_4_^2–^ also exhibited the smallest Tafel slope (65.3 mV dec^–1^) among all the tested catalysts, demonstrating the fastest reaction kinetics (Figure 2c). These results underscore both the influence of the anion and the high electrocatalytic activity of the HELDH.

**Figure 2:**
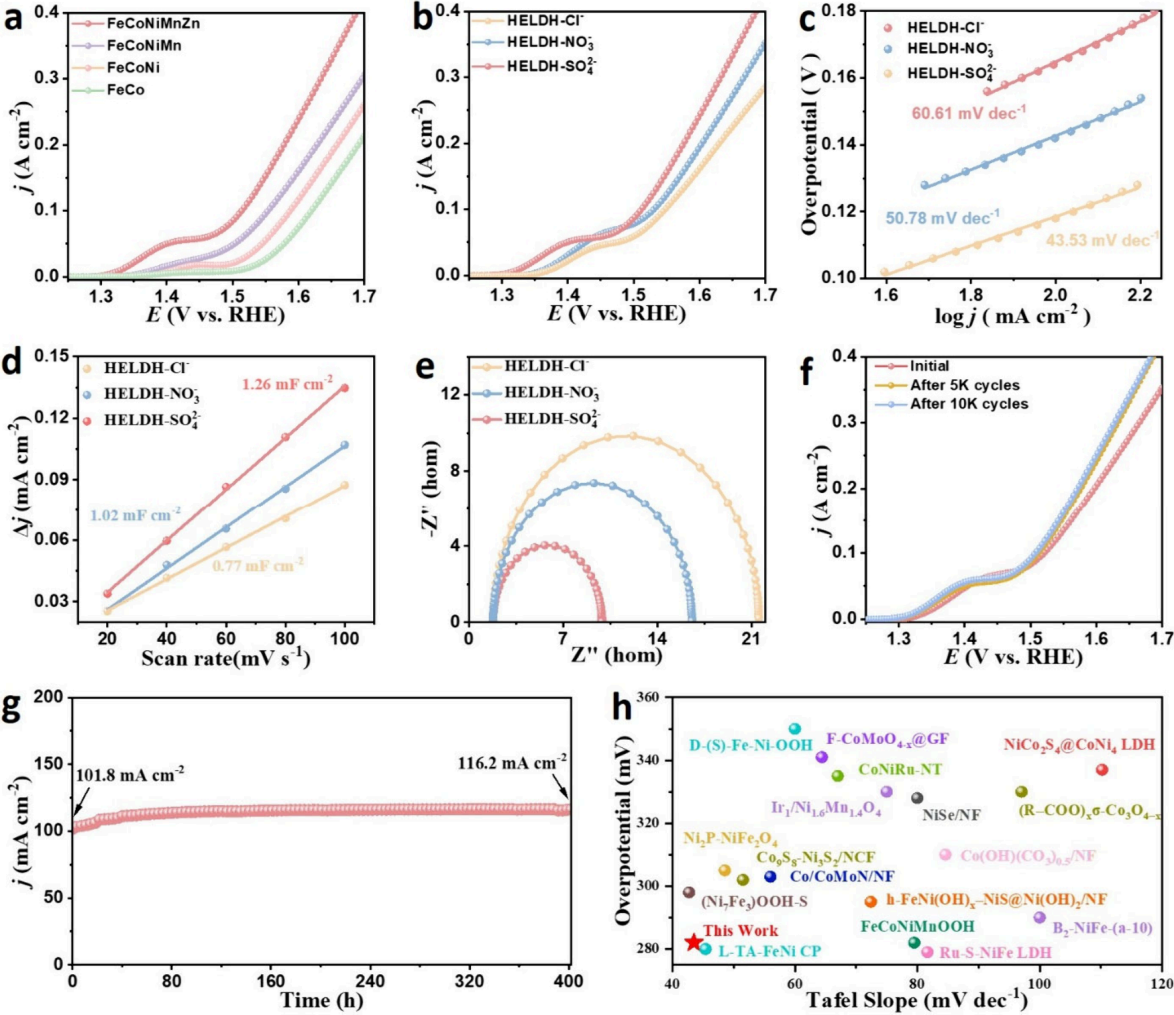
Electrocatalytic OER performance. (a) LSV curves of different metal types for LDH. (b) LSV curves, (c) Tafel plots, (d) double-layer capacitance (C_dl_), and (e) EIS Nyquist plots of HELDH-SO_4_^2–^, HELDH-NO_3_^–^, and HELDH-Cl^–^. (f) LSV curves of HELDH-SO_4_^2–^ for initial state and after 5000 and 10000 cycles. (g) Chronopotentiometry at 100 mA cm^–2^ of HELDH-SO_4_^2–^. (h) Comparison of OER overpotential at 100 mA cm^–2^ and Tafel slopes of HELDH-SO_4_^2–^ with some reported transition metal-based catalysts (Table S4).

The electrochemically active surface area (ECSA), a key indicator of the number of exposed catalytic sites, was evaluated to clarify the origin of the intrinsic OER activity enhancement in HELDH-SO_4_^2–^.^39,40^ Cyclic voltammetry (CV) was conducted in the nonfaradaic potential region at various scan rates, and the resulting double-layer capacitance (C_dl_) values were derived (Figure S10) to qualitatively estimate the ECSA in Table S2.^41,42^ As shown in Figure 2d, the ECSA values for HELDH-SO_4_^2–^, HELDH-NO_3_^–^, and HELDH-Cl^–^ were 31.5, 25.5, and 19.3 cm^2^, respectively. The superior activity of HELDH-SO_4_^2–^ can be attributed to the synergistic influence of the high-entropy effect, incorporation of sulfate ions, weak crystallinity, and its highly porous nanosheet morphology.^43,44^

Electrochemical impedance spectroscopy (EIS) was used to evaluate the interface charge transfer rate (R_ct_).^45–47^ HELDH-SO_4_^2–^ exhibits a lower R_ct_ than those of the other samples (Figure 2e, Table S3), confirming that the catalyst possesses superior charge-transfer kinetics and enhanced electronic conductivity, in line with its minimal Tafel slope.

Figure 2f shows the initial LSV curve and the LSV profiles of HELDH-SO_4_^2–^ after 5000 and 10000 CV cycles. The overpotential required to catalyze water oxidation at 100 mA cm^–2^ after 5000 and 10000 CV cycles remained virtually unchanged after prolonged cycling, which was significantly lower than that observed in the initial LSV, indicating notable activation during cycling. The chronoamperometry (i-t) durability test in Figure 2g further shows that HELDH-SO_4_^2–^ maintains a stable and even slightly increasing current density over 400 h of continuous operation, highlighting its excellent durability under alkaline water oxidation conditions.

We propose that the reduction in overpotential after CV cycling and the gradual rise in current density during chronoamperometry are associated with progressive Zn^2+^ leaching. To verify this, the dissolution behavior of different metals was examined by determining their concentrations in the electrolyte via inductively coupled plasma mass spectrometry (ICP–MS). As shown in Figure 3a, Fe, Co, Ni, and Mn remained at negligible levels throughout the 18-h test, whereas Zn content increased markedly during the first 12 h, indicating substantial leaching. This process likely generates metal vacancies and exposes additional catalytic sites, thereby boosting OER performance.^48,49^ Remarkably, the catalyst reported here exhibits OER activity among the highest for transition-metal-based systems, confirming the competitiveness of HELDH-SO_4_^2–^ (Figure 2h, Table S4).

**Figure 3:**
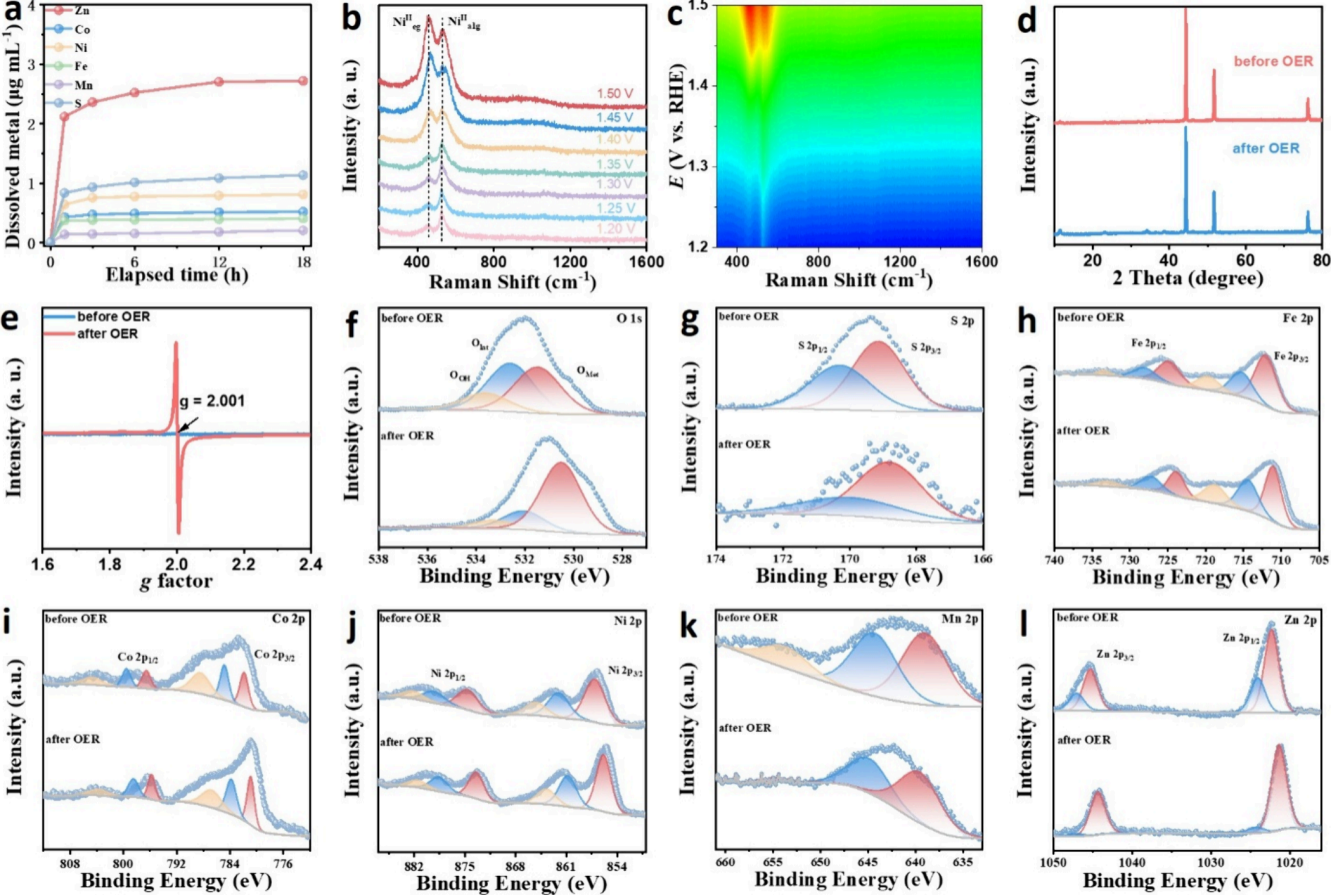
(a) Time-dependent concentration of Zn, Co, Ni, Fe, Mn, and S dissolved in the electrolyte during an 18 h chronoamperometric test. (b) In situ Raman spectra and (c) corresponding contour plots, (d) XRD, (e) EPR, and (f) O 1s, (g) S 2p, (h) Fe 2p, (i) Co 2p, (j) Ni 2p, (k) Mn 2p, and (l) Zn 2p high resolution XPS spectra before and after OER of HELDH-SO_4_^2–^.

### 2.3 Structural and Chemical Evolution

In situ electrochemical Raman spectroscopy was conducted to verify the active phase transformation from fresh HELDH-SO_4_^2–^ to FeCoNiMnZnOOH during the alkaline OER process. As shown in Figure 3b-c, increasing the applied potential from 1.20 to 1.50 V (vs RHE) led to the appearance of two initial Raman peaks at 462 and 525 cm^–1^, corresponding to the E_g_ bending vibration (δ­(Ni–O)) and A_1g_ stretching vibration (υ­(Ni–O)) modes in Ni–O, respectively.^12,14,50,51^ With increasing potential, both peaks are exhibited a blue-shift and transitioned from Ni^II^–O to Ni^III^–O, indicating the progressive oxidation of Ni and the structural reconstruction of the catalyst into FeCoNiMnZnOOH under OER conditions. After OER, XRD, and HRTEM analyses reveal the emergence of a new crystalline phase formed during operation (Figure 3d and Figure S11d–e). SEM and TEM images confirm that the ultrathin nanosheet morphology is largely preserved after the OER durability test (Figure S11a–c). HAADF-STEM images together with EELS elemental maps (Figure S11f–h) show a generally homogeneous elemental distribution, while the Zn signal is noticeably attenuated, consistent with partial Zn leaching. In addition, electron paramagnetic resonance (EPR) spectroscopy (Figure 3e) provides further evidence for the formation of cation defect sites induced by Zn leaching.

To further understand the origins of the excellent OER catalytic performance, the surface composition and chemical states of HELDH-SO_4_^2–^ before and after the OER test were examined using X-ray photoelectron spectroscopy (XPS). The high-resolution O 1s XPS spectrum before OER (Figure 3f) displays two peaks at 532.6 and 531.5 eV, corresponding to lattice oxygen (O_lat_) and surface metal hydroxyl groups (O_Met_), respectively. After OER, a negative shift in the O_lat_ binding energy is observed, attributed to the formation of metal oxyhydroxide species that increase the local electron density around oxygen atoms and alter their chemical environment.

Figure 3g shows a clear S 2p XPS signal associated with sulfur within a sulfate chemical environment (S 2p_3/2_ = 169.1 eV), both before and after the OER test. The Fe 2p spectrum (Figure 3h) exhibits six peaks, corresponding to Fe^2+^ (712.1/724.8 eV), Fe^3+^ (715.3/728.1 eV), and two satellite features at 719.7 and 733.4 eV. After OER, these peaks shift by approximately +0.8 eV, indicating a slight increase in the oxidation state of Fe.^52,53^ Similarly, the Co 2p spectrum (Figure 3i) shows peaks attributed to Co^2+^ (781.8/796.5 eV) and Co^3+^ (784.8/799.4 eV). These also exhibit positive shifts, suggesting additional oxidation during the reaction.^21,54,55^ The Ni 2p_3/2_ spectrum (Figure 3j) features peaks at 856.9 and 862.1 eV for Ni^2+^ and Ni^3+^, respectively, both of which shift by +0.8 eV after OER, indicating reduced electron density at Ni sites.^8,46^ In the Mn 2p region (Figure 3k), peaks at 641.4 and 646.3 eV correspond to Mn^3+^ and Mn^4+^, respectively, with the latter increasing after OER, reflecting a higher Mn oxidation state.^56^ Notably, the XPS signals of Zn became weak after OER (Figure 3l), consistent with the Zn leaching in an alkaline medium observed by ICP-MS.^22^ This selective leaching is expected to generate cation vacancies, thereby enhancing catalytic activity by exposing additional active sites and modulating the local electronic structure.

### 2.4 DFT Calculations

Density functional theory (DFT) calculations were conducted to explore the impact of adsorbed sulfate species and zinc leaching on the catalytic activity and stability toward the OER. Based on the in situ Raman results, three computational models were constructed: FeCoNiMnZnOOH, FeCoNiMnZnOOH with a zinc vacancy (FeCoNiMnZnOOH-V_c,_), and the sulfate-adsorbed system (SO_4_^2–^-FeCoNiMnZnOOH-V_c,_). The (100) facet was selected as the representative active surface based on the HRTEM observations after OER and prior reports indicating that this facet commonly shows higher OER activity.^19,57^ To simplify the calculations, we then chose Ni sites adjacent to the defect region as the computational active sites, guided by the in situ Raman results and the Bader charge analysis (Figure S12).

The OER on the SO_4_^2–^-FeCoNiMnZnOOH-V_c_ surface comprises four proton-coupled electron transfer steps and involves three main intermediates: *OH, *O, and *OOH, as shown in Figure 4a. The impact of adsorbed sulfate and zinc leaching on the electronic structure was first examined through density of states (DOS) analysis. As shown in Figure 4d, the total DOS of the FeCoNiMnZnOOH, Zn-deficient FeCoNiMnZnOOH (FeCoNiMnZnOOH-V_c_), and the adsorbed sulfate group and zinc-deficient system (SO_4_^2–^-FeCoNiMnZnOOH-V_c_) exhibit distinct features, particularly near the Fermi level. SO_4_^2–^-FeCoNiMnZnOOH-V_c_ displays a noticeable increase in the DOS in the vicinity of the Fermi level, indicating enhanced electronic conductivity, enhanced charge transfer capability, and thus a more favorable electronic structure for the adsorption and activation of OER intermediates.

**Figure 4:**
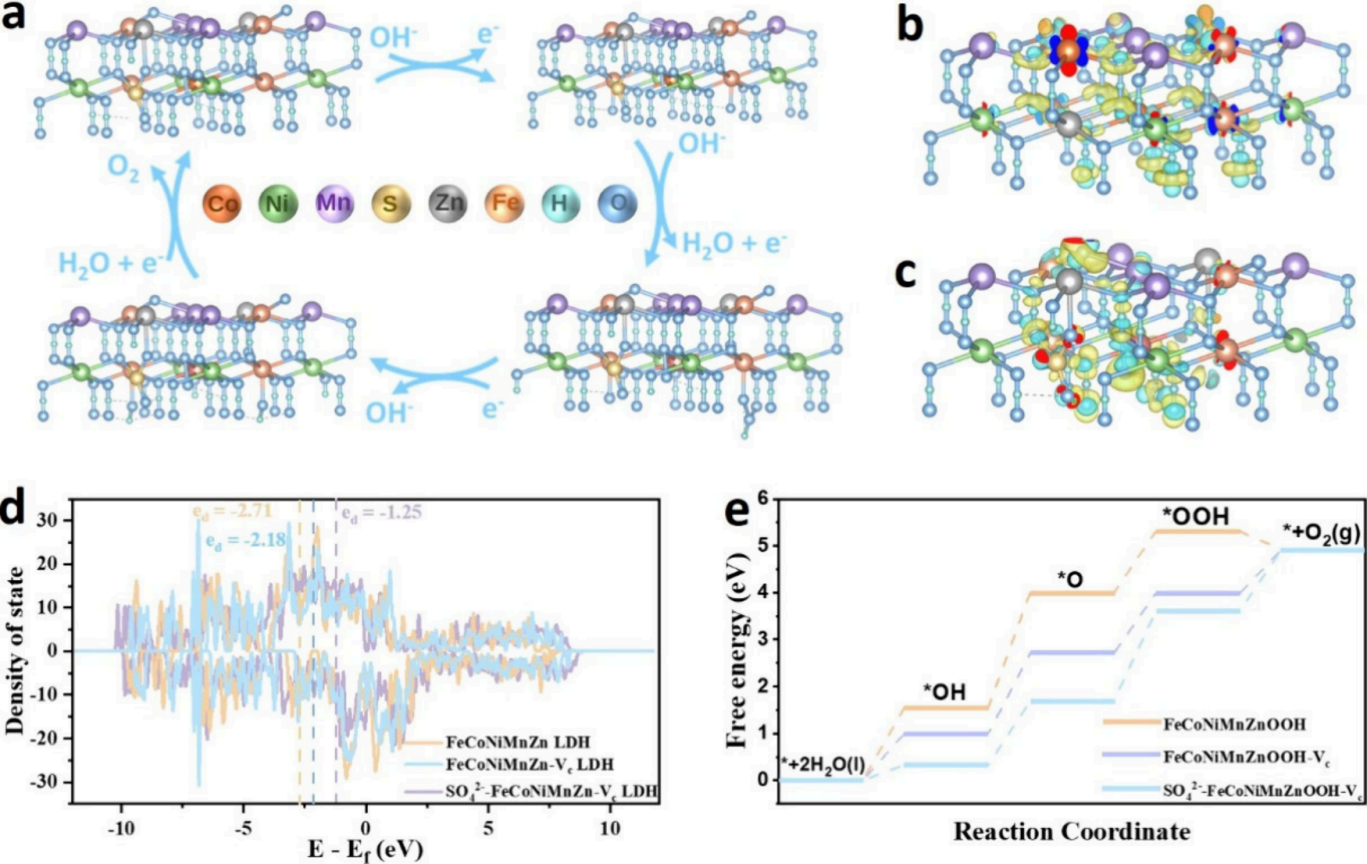
(a) Reaction pathway diagram of OER on SO_4_^2–^-FeCoNiMnZnOOH-V_c_. (b, c) Charge density difference maps of FeCoNiMnZnOOH and SO_4_^2–^-FeCoNiMnZnOOH-V_c_, (d) DOS, and (e) OER Gibbs free energy profiles for FeCoNiMnZnOOH, FeCoNiMnZnOOH-V_c,_ and SO_4_^2–^-FeCoNiMnZnOOH-V_c_, respectively.

The detailed Gibbs free energy profiles for the OER pathways are shown in Figure 4e. In transition metal (oxy)­hydroxides, the formation of *O intermediates is commonly the rate-determining step (RDS), due to the high energy required for O–H bond cleavage and deprotonation.^19,41^ This trend is also observed in FeCoNiMnZnOOH and FeCoNiMnZnOOH–V_c_, where *O formation presents the highest energy barrier, confirming its role as the RDS. The corresponding energy barriers are 2.45 eV for FeCoNiMnZnOOH and 1.71 eV for FeCoNiMnZnOOH–V_c_, indicating that the presence of cation vacancies, introduced by Zn leaching, facilitates oxygen intermediate binding and significantly enhances OER kinetics. Interestingly, for SO_4_^2–^-FeCoNiMnZnOOH-V_c_, the RDS shifts from *O formation to *O desorption, highlighting the critical role of sulfate adsorption in modulating the reaction pathway.

The differential charge densities of FeCoNiMnZnOOH, SO_4_^2–^-FeCoNiMnZnOOH-V_c_ and FeCoNiMnZnOOH-V_c,_ are presented in Figures 4b-c and S13, respectively. It is evident that the adsorbed sulfate groups and defect sites formed after Zn leaching favor the stabilization of *OH, thereby facilitating its deprotonation. These results suggest that defect sites act as adsorption centers for *O, while metal sites preferentially bind other OER intermediates, in agreement with previous reports.^19,20^

### 2.5 AEMWE Devices

Motivated by the superior OER catalytic performance of HELDH-SO_4_^2–^, AEMWEs were assembled using this catalyst as the anode to evaluate its practical application potential in water splitting. Commercial platinum carbon was sprayed onto an AEM to be used as cathode and separator. For comparison, a commercial Pt/C (cathode) and IrO_2_ (anode) electrolyzer was also tested under identical conditions.

The Pt/C∥HELDH-SO_4_^2–^ electrolyzer exhibited a remarkably low cell voltage of 1.84 V at a current density of 1.0 A cm^–2^ at 60 °C (Figure 5b). When the temperature was increased to 80 °C, the enhanced mobility of OH^–^ ions within the AEM further reduced the cell voltage to 1.78 V at the same current density, which is the lowest value reported (Table S2). This value also significantly outperforms that of the Pt/C∥IrO_2_ electrolyzer, which required 1.82 V to drive 1 A cm^–2^ at 80 °C, indicating exceptional catalytic activity for alkaline water splitting (Figure 5c). In terms of durability, the Pt/C∥HELDH-SO_4_^2–^ electrolyzer demonstrated exceptional stability, with no significant voltage degradation observed after 200 h of continuous operation under stepwise current densities of 1 A cm^–2^ (Figure 5d). The superior performance of AEMWE may origin from the self-grown ultrathin nanosheet architecture of HELDH-SO_4_^2–^ that provided a high specific surface area and open transport pathways, enabling efficient active-site utilization and mitigating mass-transport limitations, which together contribute to the low cell voltage and stable durability observed in AEMWE operation. These findings highlight the outstanding performance of HELDH-SO_4_^2–^ as the anode catalyst in an AEMWE, demonstrating its practical application potential for efficient and durable alkaline water splitting.

**Figure 5:**
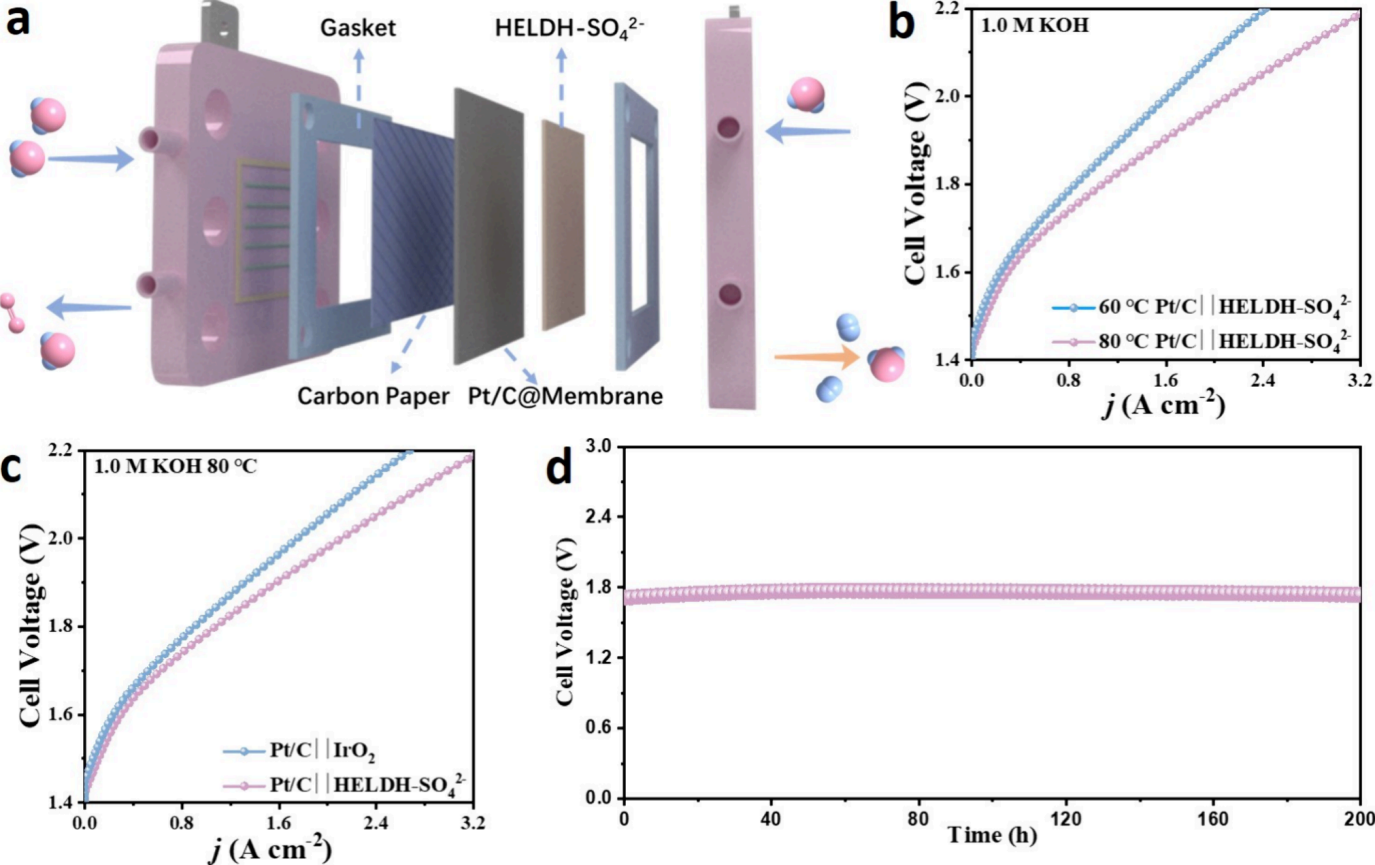
(a) Schematic image of the AEM electrolyzer. (b) Polarization curves of the AEMWE employing Pt/C∥HELDH-SO_4_^2–^ couples at different temperatures. (c) Performance of the AEMWE employing Pt/C∥IrO_2_ and Pt/C∥HELDH-SO_4_^2–^ couples at 80 °C. (d) Durability test of the AEMWE employing Pt/C∥HELDH-SO_4_^2–^ at 80 °C under 1000 mA cm^–2^.

## 3 Conclusion

In summary, the influence of several anions (SO_4_^2–^, NO_3_^–^, Cl^–^) on the structural, chemical, and functional properties of HELDH electrocatalyst was analyzed. Among them, HELDH-SO_4_^2–^ demonstrated outstanding OER performance, including a low Tafel slope of 45.53 mV dec^–1^ and a small overpotential of 282 mV at 100 mA cm^–2^, which is attributed to the formation of catalytically active cation vacancies induced by the selective leaching of Zn^2+^ ions and adsorbed sulfate under alkaline conditions. DFT calculations proved that the SO_4_^2–^ decoration and the defects generated by zinc etching facilitate the O* desorption and lower the reaction energy barrier, thus accelerating the OER process. Moreover, using HELDH-SO_4_^2–^ as the anode, the as-fabricated AEMWE exhibited a high performance with a cell voltage of 1.78 V and outstanding stability exceeding 200 h. This work not only provides insights into anion-directed morphological engineering and leaching strategy, but also presents HELDHs as promising materials to develop next-generation OER catalysts for sustainable hydrogen production.

## 4 Experimental Section

High entropy layered hydroxides: A piece of nickel foam (NF, 2 × 3 cm) was washed in anhydrous ethanol and DIW, sonicated in a 3 M HCl solution for 15 min to clean the surface. Subsequently, 0.5 mmol FeSO_4_·7H_2_O, 0 CoSO_4_·7H_2_O, NiSO_4_·7H_2_O, NiSO_4_·4H_2_O, ZnSO_4_·7H_2_O, and 10 mmol urea were added to 80 mL of DIW solution and stirred for 30 min. Afterward, the mixed solution with the pretreated NF was transferred to a 100 mL Teflon-lined stainless-steel autoclave and kept at 120 °C for 10 h. The obtained NF was washed with DIW and dried at 60 °C, and named as HELDH-SO_4_^2–^. HELDH-SO_4_^2–^, HELDH-Cl^–^ were prepared using the same method by using same molar amount metal nitrates (0.5 mmol Fe­(NO_3_)_3_·9H_2_O, Co­(NO_3_)_2_·6H_2_O, Ni­(NO_3_)_2_·6H_2_O, Mn­(NO_3_)_2_·6H_2_O, Zn­(NO_3_)_2_·6H_2_O) and metal chlorides (0.5 mmol FeCl_3_·6H_2_O, CoCl_2_·6H_2_O, NiCl_2_·6H_2_O, MnCl_2_·4H_2_O, ZnCl_2_) to replace metal sulfates.

## Supplementary Material



## Notes

Chemical details and structural characterization method; details for electrochemical measurements for anion exchange membrane water electrolysis (AEMWE) and theoretical modeling and calculations; SEM, TEM, HRTEM, and HAADF-STEM images; SAED pattern, AFM images; XRD patterns; FTIR spectra; BET isotherms; CV curves; Bader charge analysis; and charge density difference maps of prepared catalysts (PDF)
