# Supplementary material for: Anion-Directed Engineering of High-Entropy Layered Double Hydroxides for Oxygen Evolution Catalysis
Source: ACS Nano. 2026 Feb 20;20(8):6732–41. doi: 10.1021/acsnano.5c15605 (PMC12961937; doi:10.1021/acsnano.5c15605)
Supplement: Supplementary file 1 [file nn5c15605_si_001.pdf]

Supporting Information

Anion-Directed Engineering of High-Entropy Layered Double Hydroxides for Oxygen Evolution Catalysis

Yin Liu,[†, ‡, #] Xiaorong Jiao,[†, ‡, #] Xingmao Jiang,[†, ‡] Congcong Xing,[§, ⊥] Xueqiang Qi,[§, ¶] Xiang Wang,[†, ‡, §]* Andreu Cabot,[§, //]*

[†] School of Chemical Engineering and Pharmacy, Hubei Key Laboratory of Novel Reactor and Green Chemical Technology, Wuhan Institute of Technology, Wuhan 430205, China.

[‡] State Key Laboratory of Green and Efficient Development of Phosphorus Resources, Wuhan 430205, China.

[§] Catalonia Institute for Energy Research (IREC), Sant Adrià de Besòs, 08930 Barcelona, Catalonia, Spain.

[⊥] Institute of Wenzhou, Zhejiang University, 325006 Wenzhou, China.

[¶] School of Chemistry and Chemical Engineering, Chongqing University, Chongqing 400044, China.

[//] ICREA, Pg. Lluis Companys 23, 08010 Barcelona, Spain.

[#] Author contributions are equal

* E-mails: X.Wang: wangxiang@wit.edu.cn; A. Cabot: acabot@irec.cat.

Experimental Section

Chemicals

Iron(III) nitrate nonahydrate ($Fe(NO_3)_2{\cdot}9H_2O$, ≥99%), cobalt(II) nitrate hexahydrate ($Co(NO_3)_2{\cdot}6H_2O$, ≥98%), nickel(II) nitrate hexahydrate ($Ni(NO_3)_2{\cdot}6H_2O$, ≥98%), manganese(II) nitrate tetrahydrate ($Mn(NO_3)_2{\cdot}6H_2O$, ≥99%), zinc nitrate hexahydrate ($Zn(NO_3)_2{\cdot}6H_2O$, ≥99%), iron chloride hexahydrate ($FeCl_3{\cdot}6H_2O$, ≥99%), cobalt(II) chloride hexahydrate ($CoCl_2{\cdot}6H_2O$, ≥99%), nickel(II) chloride hydrate ($NiCl_2{\cdot}6H_2O$, ≥99%), manganese(II) chloride tetrahydrate ($MnCl_2{\cdot}4H_2O$, ≥98%), zinc chloride ($ZnCl_2$, ≥98%), ferrous sulfate heptahydrate ($FeSO_4{\cdot}7H_2O$, ≥99%), cobalt sulfate heptahydrate ($CoSO_4{\cdot}7H_2O$, ≥99%), nickel(II) sulfate heptahydrate ($NiSO_4{\cdot}7H_2O$, ≥99%), manganese sulfate tetrahydrate ($MnSO_4{\cdot}7H_2O$, ≥99%), zinc sulfate heptahydrate ($ZnSO_4{\cdot}7H_2O$, ≥99%), iron chloride hexahydrate ($FeCl_3{\cdot}6H_2O$, ≥99%), cobalt(II) chloride hexahydrate ($CoCl_2{\cdot}6H_2O$, ≥99%), nickel(II) chloride hydrate ($NiCl_2{\cdot}6H_2O$, ≥99%), manganese(II) chloride tetrahydrate ($MnCl_2{\cdot}4H_2O$, ≥99%), zinc chloride ($ZnCl_2$, ≥99%), urea ($CO(NH_2)_2$, ≥95%), and potassium hydroxide (KOH, 85%) were purchased from Adamas. Nafion (5 wt% in a mixture of low aliphatic alcohols and water) was obtained from Aladdin. The reference Pt catalyst, 20% Pt on activated carbon powder, was purchased from Alfa Aesar. Ethanol was of analytical grade and obtained from various sources. Deionized water (DIW) was obtained from a Purelab flex from Elga. All chemicals were used as received, without further purification.

Structural characterization

Powder X-ray diffraction (XRD) was performed on a Bruker AXS D8 Advance X-ray diffractometer with Cu-Kα radiation (λ= 1.5406 Å). Scanning electron microscopy (SEM) analysis was conducted with a Zeiss Auriga microscope equipped with an energy dispersive spectroscopy (EDS) detector operating at 20 kV. Transmission electron microscopy (TEM), high-resolution TEM (HRTEM), annular dark-field scanning transmission electron microscope (HAADF-STEM), and electron energy loss spectroscopy (EELS) analysis were performed on a field emission gun FEI™ Tecnai F20 microscope with a Gatan Quantum filter at 200 kV. X-ray photoelectron spectroscopy (XPS) measurements were conducted on a SPECS system equipped with an Al anode XR50 source operating at 150 W and a Phoibos 150 MCD-9 detector. Inductively coupled plasma mass spectrometry (ICP-MS) was conducted with a Shimadzu

simultaneous ICP atomic emission spectrometer ICPE-9820.

Electrochemical measurements

The electrochemical performance was evaluated in 1.0 M KOH using a CHI760E electrochemical workstation. Hg/HgO (1 M KOH) and graphite rods were employed as reference and counter electrodes, respectively. The working electrode was the as−prepared electrocatalyst supported on NF with an area of 1.0 cm^2. Before tests, O_2 flowed through the electrolyte for about 30 min to achieve a saturated solution. Linear sweep voltammetry (LSV) tests were measured in O_2-saturated solution with a scan rate of 5 mV/s. Cyclic voltammetry (CV) was conducted in O_2-saturated electrolyte at different scan rates of 20 to 100 mV s^{-1}. Electrochemical impedance spectroscopy (EIS) was measured in a frequency range from 100 kHz to 0.01 Hz at 5 mV. In situ Raman spectra were collected by using a Horiba Jobin-Yvon LabRAM HR800 spectrometer with a 514 nm laser. The three-electrode system used Ag/AgCl as the reference electrode, platinum wire as the counter electrode, and 1.0 M KOH as the electrolyte.

Anion exchange membrane water electrolysis (AEMWE)

An AEMWE cell was used to evaluate the water-splitting performance of the synthesized electrocatalysts. In the membrane electrode assembly (MEA), an anion exchange membrane (Sustanion® x37-50) separated the cathode and anode compartments. HELDH (1 cm × 1 cm) served as anode and Commercial Pt/C as cathode. For comparison, commercial RuO_2 was used as the anode catalysts. During water splitting, 1 M KOH was continuously supplied to both sides of the electrolyzer at 60°C and 80°C. Polarization curves were recorded by stepping the current density up to 3200 mA cm^{-2} at a scan rate of 10 mV s^{-1}. The stability of the AEMWE was assessed via CP at 1000 mA cm^{-2} for 200 h.

Theoretical modelling and calculations

All spined calculations were performed in the framework of the density functional theory (DFT) with the projector augmented planewave method (PAW), by using the Vienna ab initio (VASP) software. The Perdew-Burke-Ernzerhof and the generalized gradient approximation were employed as the functional form and the description of the electron exchange and associated energies, respectively. For calculation parameters, the cut-off energy was set to 450 eV, and the convergence criteria for force and energy were set at 0.01 eV $Å^{-1}$ and 10−5 eV, respectively.

Using the Monkhorst-Pack scheme of (3 × 3 × 1), sampling of the Brillouin zone was carried out for all model optimizations, and all plates were added with a 15 Å vacuum layer to separate their periodicity. For all calculations of density of states, the K point of the Brillouin zone is taken as (9 × 9 × 1). The Hubbard-U correction (DFT + U method) was applied to improve the description of localized metal d-electrons in the FeCoNiMnZnOOH, FeCoNiMnZnOOH-V_c and SO_4^{2-}-FeCoNiMnZnOOH-V_c systems. the value of U was set as 3.9, 4.0, 3.3, 6.0 and 3.6 eV for Mn, Fe, Co, Ni and Zn, respectively. The Gibbs free energy change was obtained based on the widely accepted OER four-electron transfer mechanism according to the previous report.

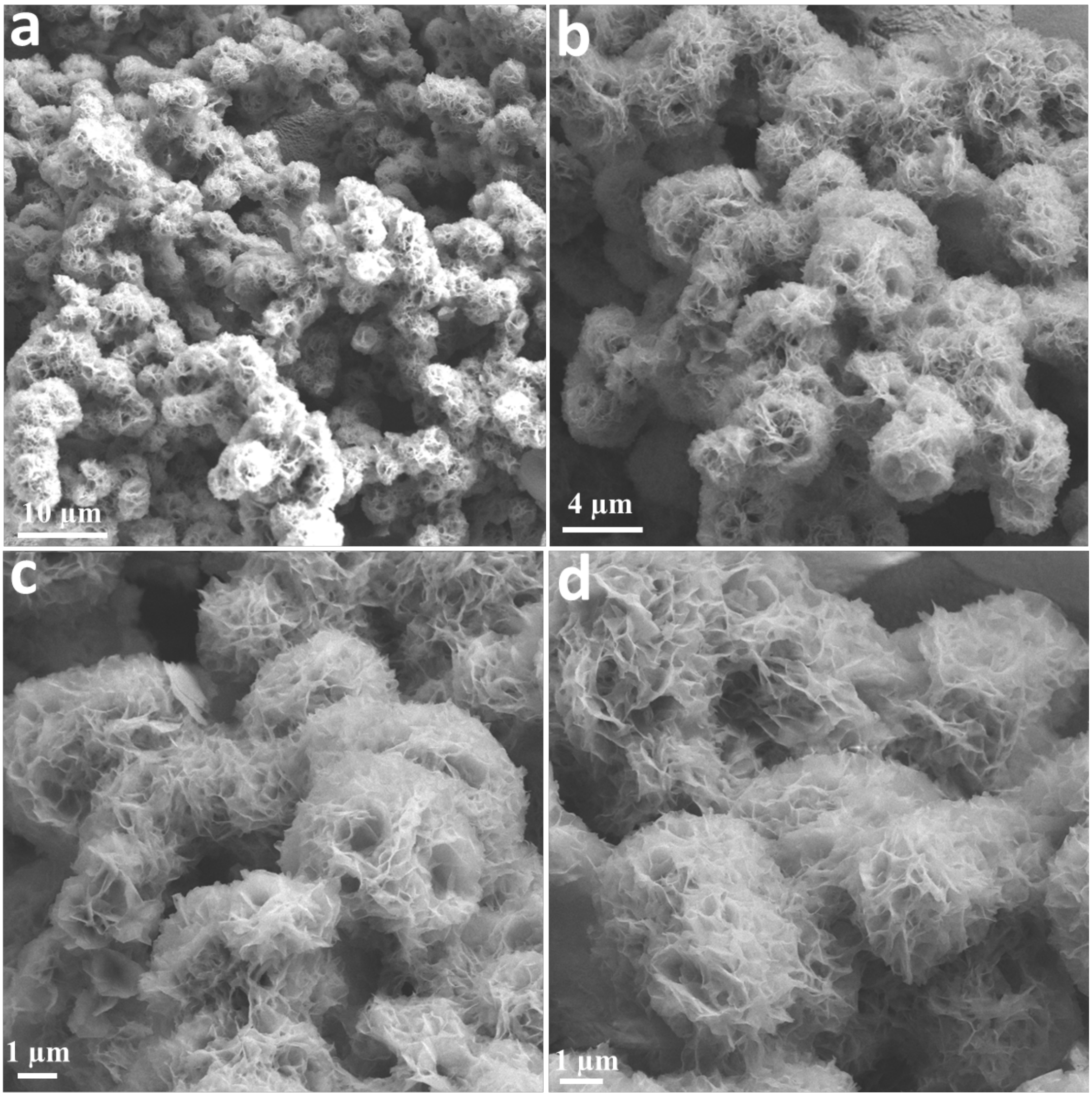

Figure S1. SEM images of HELDH-SO_4^{2-}

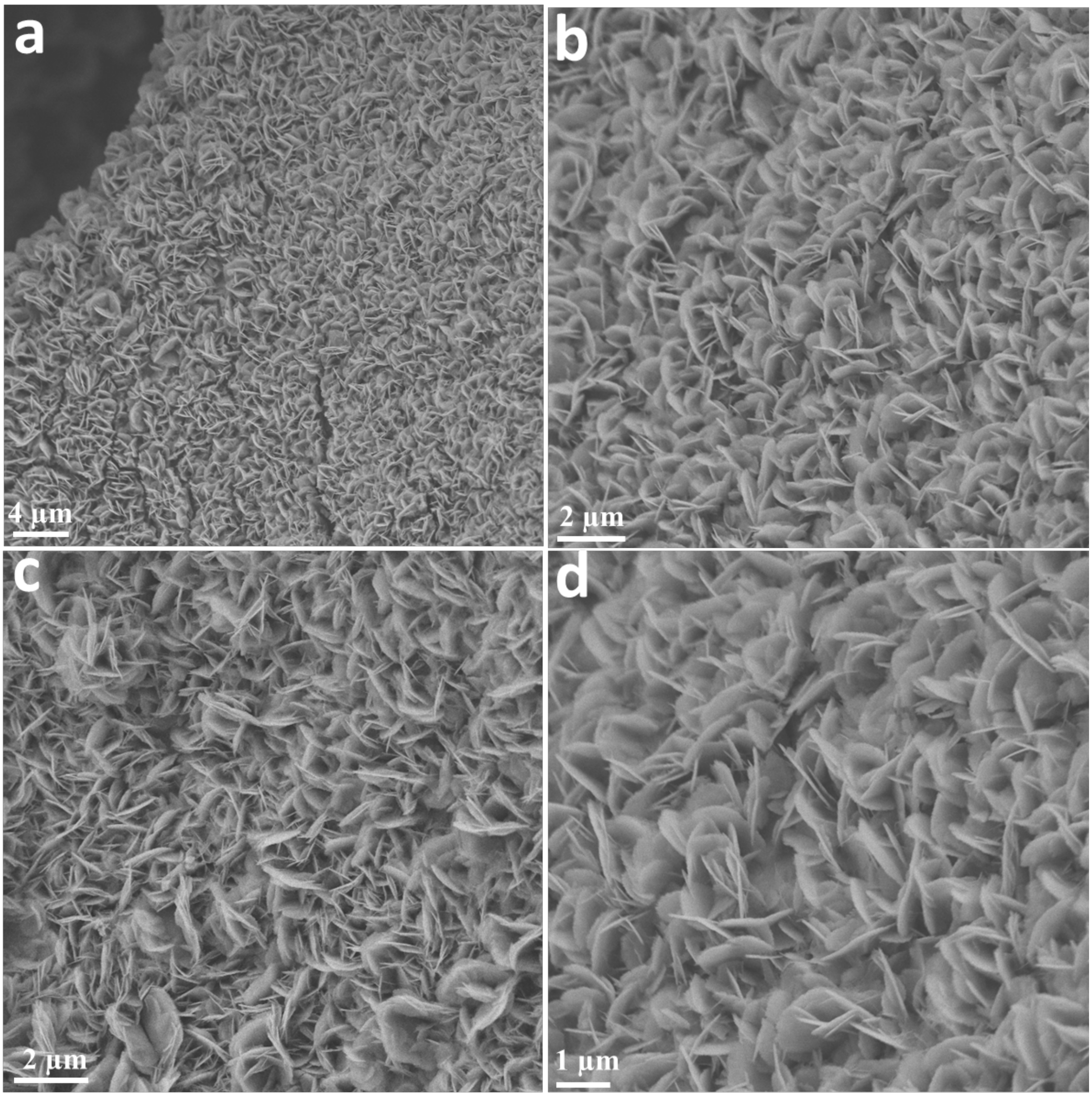

Figure S2 SEM images of HELDH-NO_3^-, HELDH-Cl^-

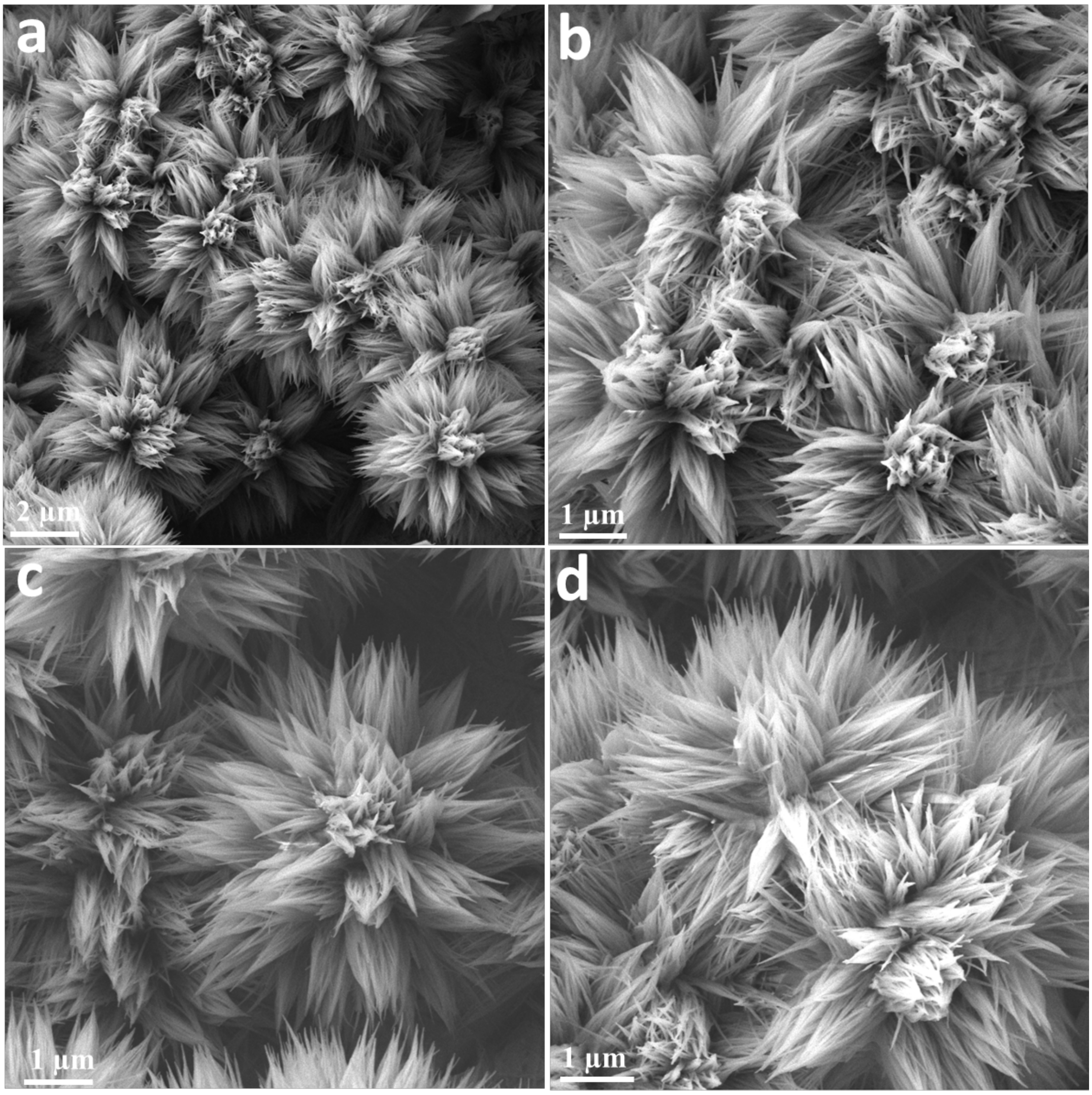

Figure S3 SEM images of HELDH-Cl^{-}

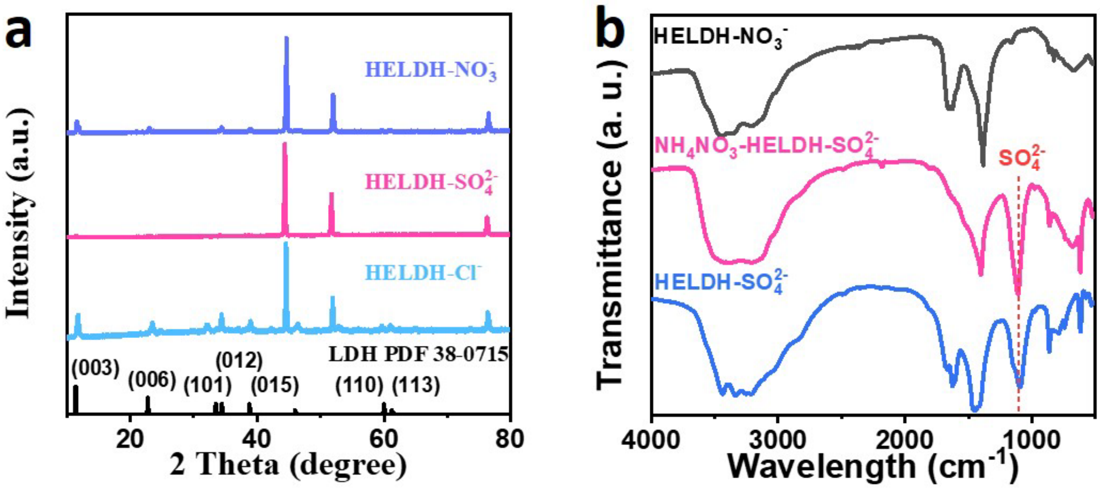

Figure S4. XRD and FTIR patterns of the as-prepared samples

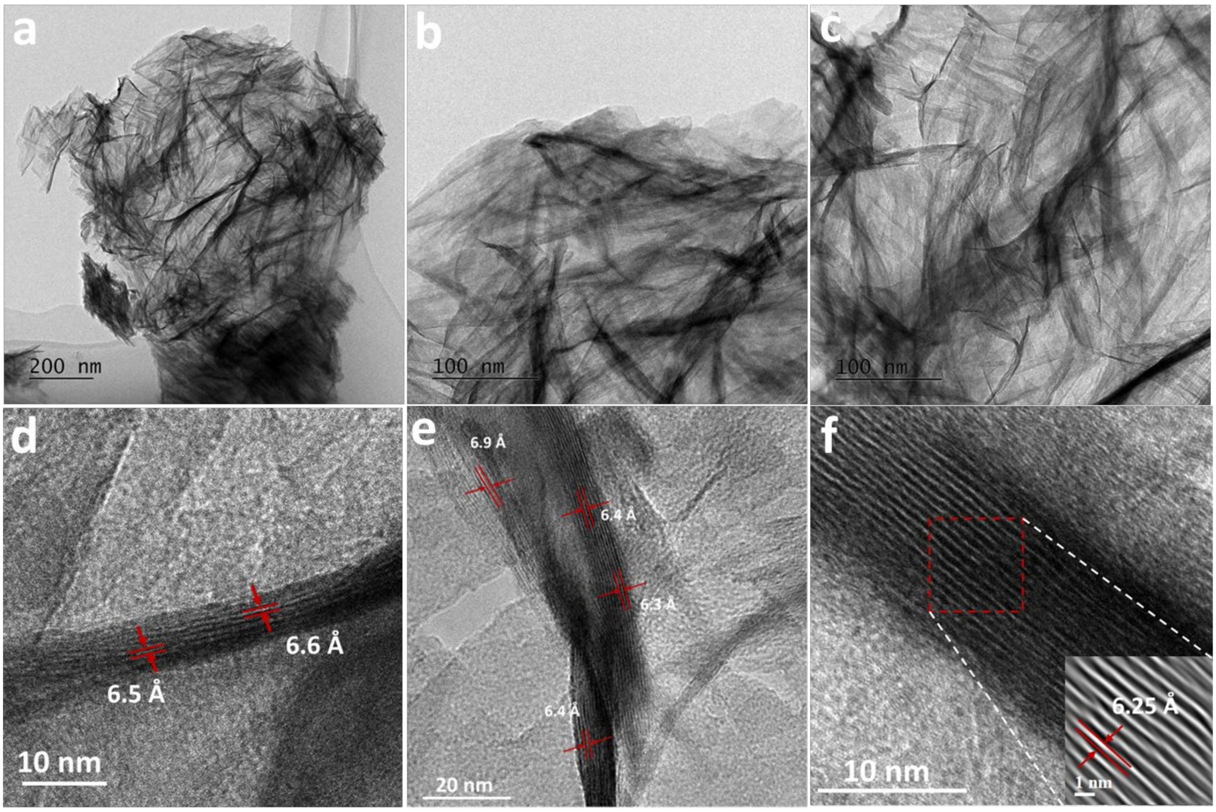

Figure S5. a-c) TEM images, d-f) HRTEM images on the edge of HELDH-SO_4^{2-}

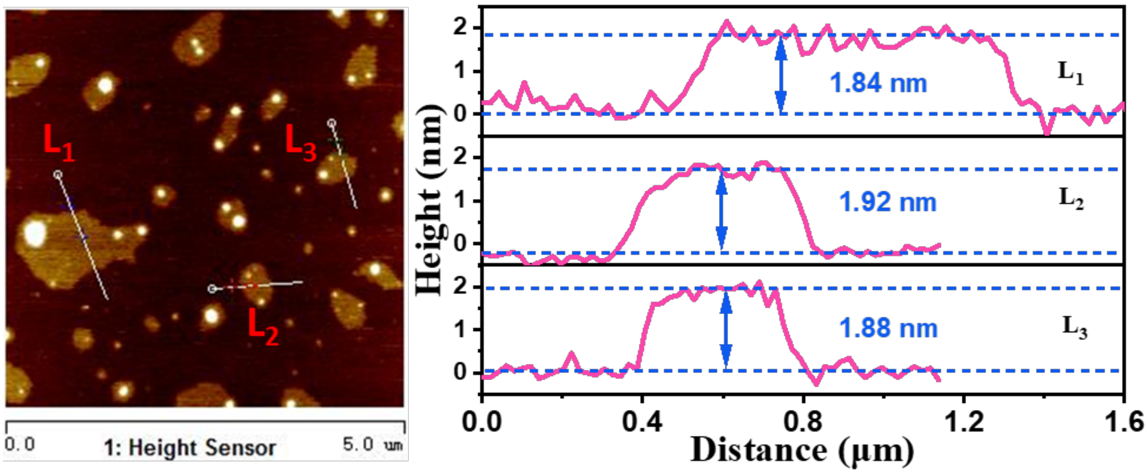

Figure S6. AFM images and the corresponding thickness measurement data of HELDH-SO_4^{2-}.

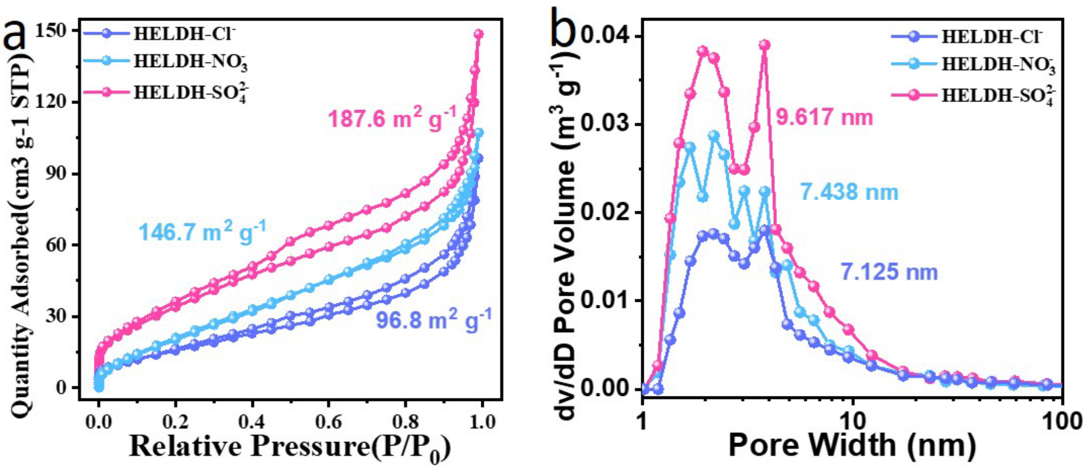

Figure S7. N_2 adsorption-desorption isotherms and pore size distribution curves of the prepared catalysts

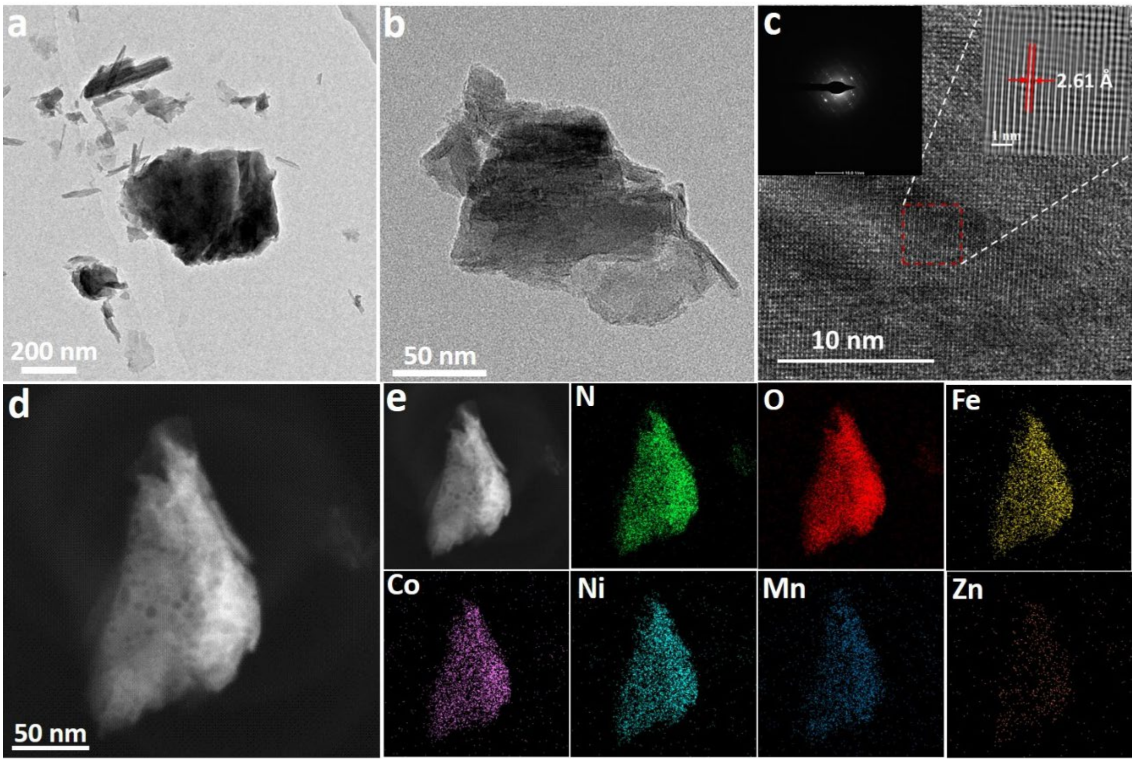

Figure S8 a-b) TEM, c) HRTEM images, d-e) HAADF-STEM images and elemental mapping images of HELDH-NO_3^-. The inset in (c) shows the corresponding SAED pattern.

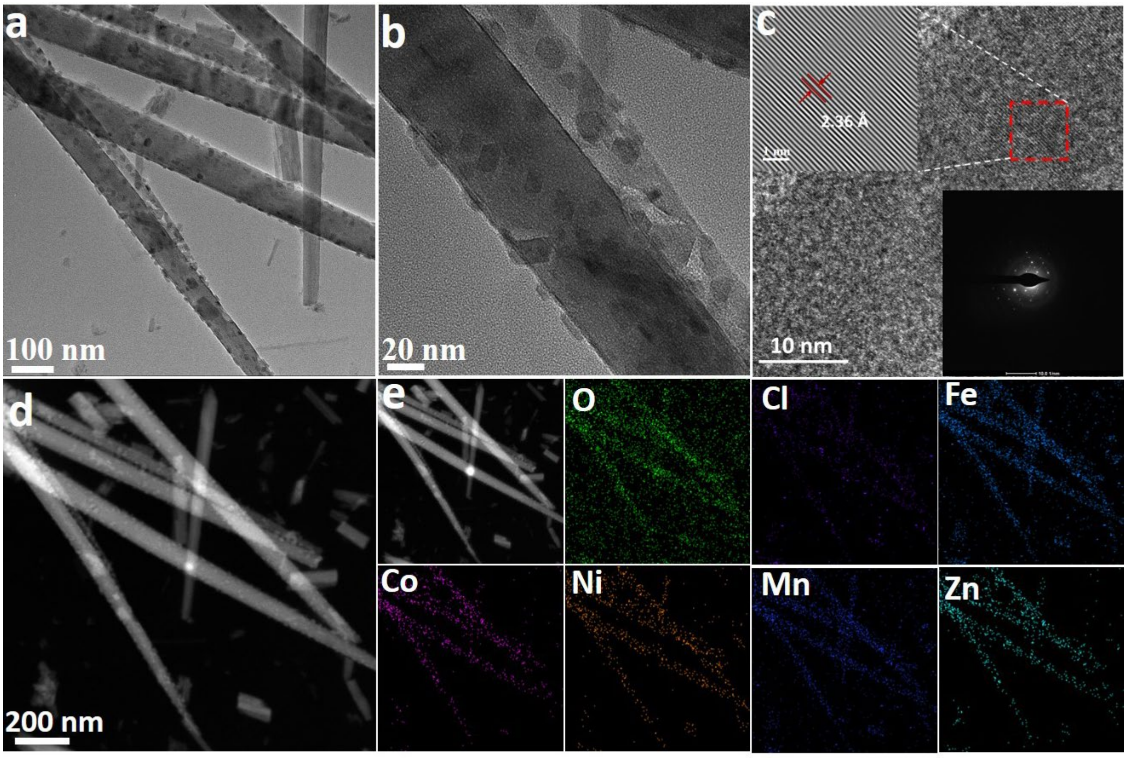

Figure S9 a-b) TEM, c) HRTEM images, d-e) HAADF-STEM images and elemental mapping images of HELDH-Cl$^-$. The inset in (c) shows the corresponding SAED pattern.

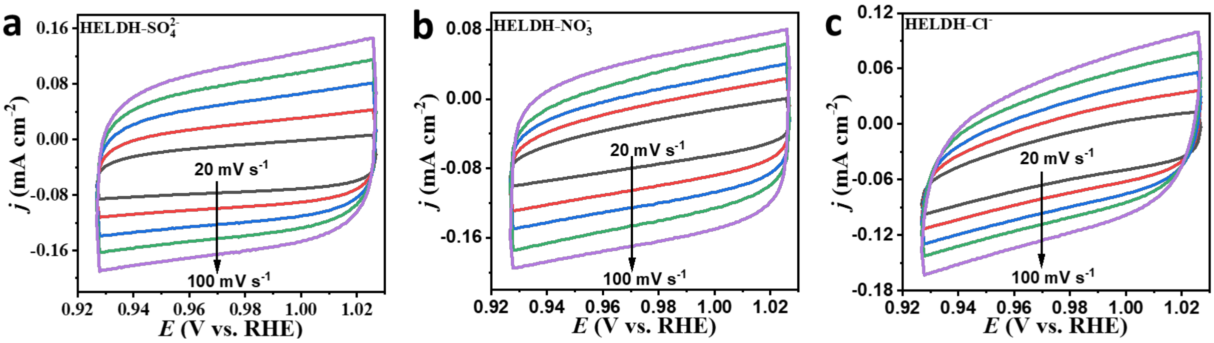

Figure S10. Cyclic voltammograms of a) HELDH-SO_4^{2-}, b) HELDH-NO_3^-, c) HELDH-Cl^- in the non-faradaic region of 0.93-1.03 V vs. RHE at various scan rates.

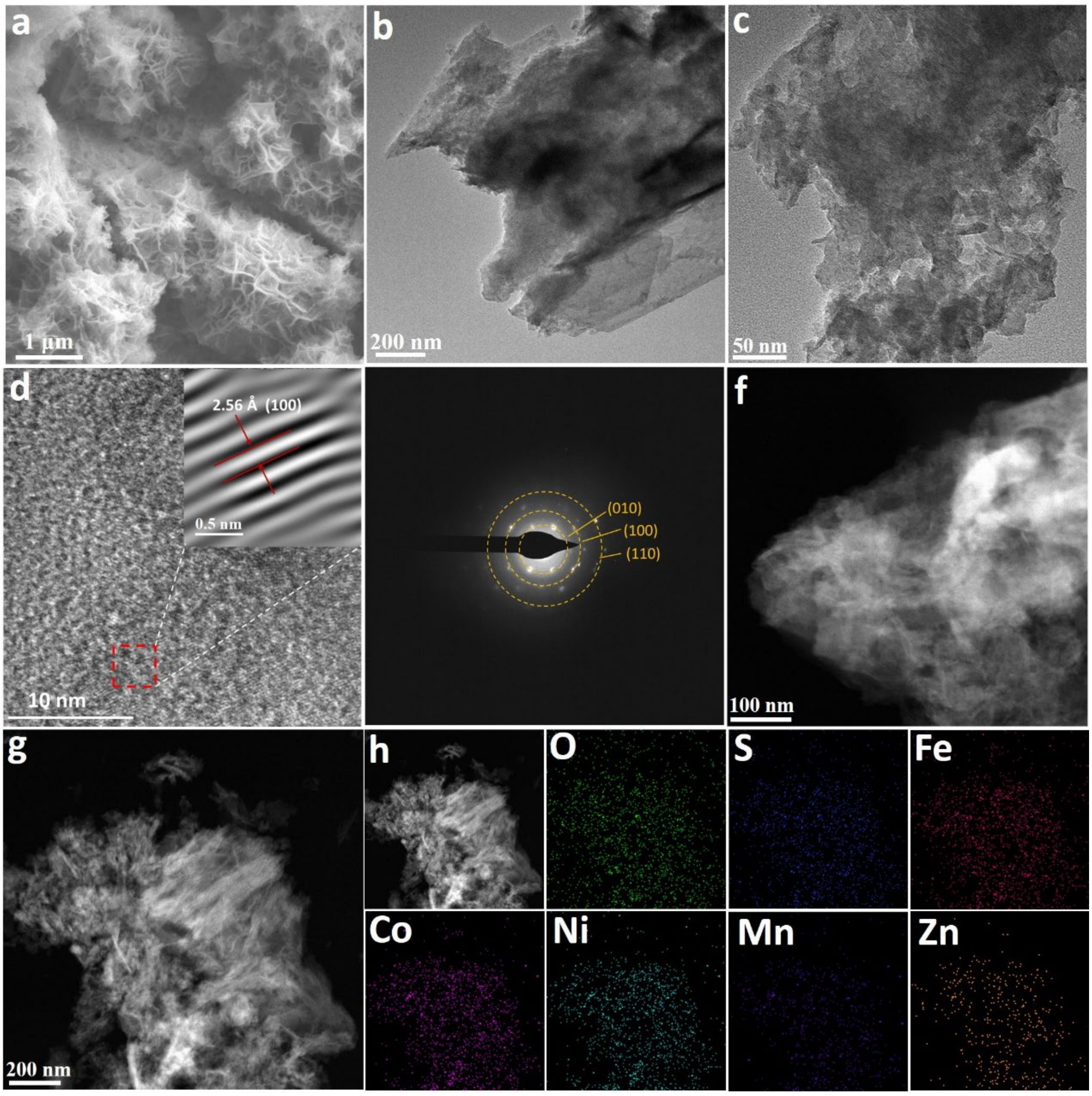

Figure S11 a) SEM, b-c) TEM, d) HRTEM images, e) corresponding SAED pattern, and f-i) HAADF-STEM images and elemental mapping images of HELDH-SO_4^{2-} after OER

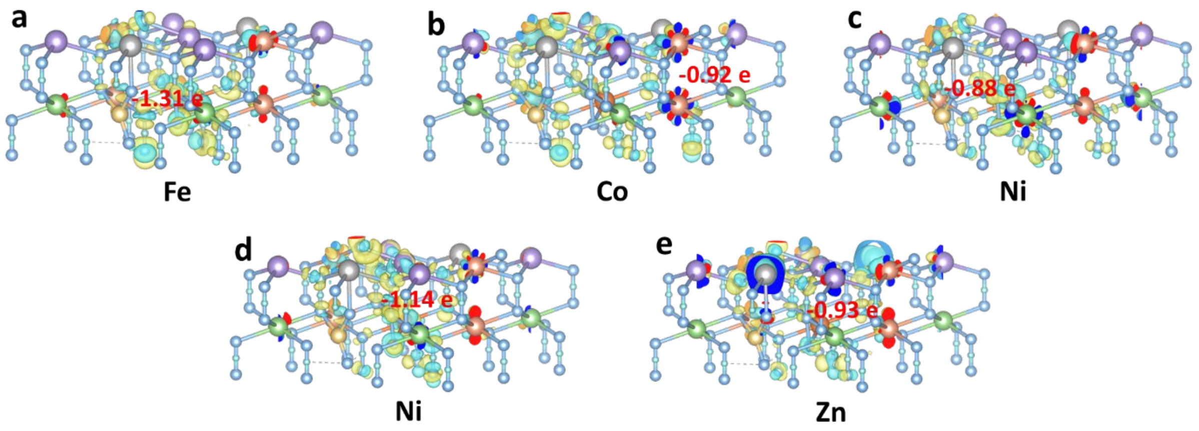

Figure S12 a-e) Bader charge analysis for Fe, Co, Ni, Mn, and Zn metal elements in SO_4^{2-}-FeCoNiMnZnOOH-V_c

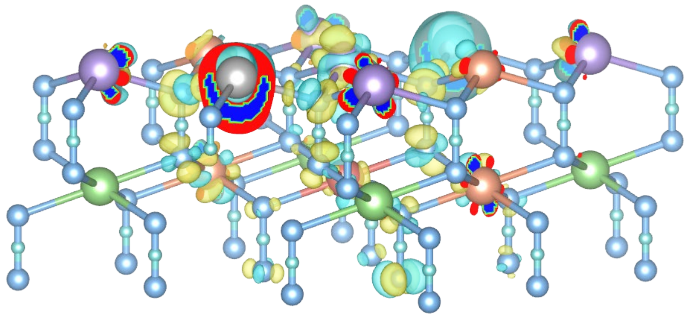

Figure S13. Charge density difference maps of FeCoNiMnZnOOH-V_c

Table S1. ICP-OES result of prepared HELDH catalysts

Catalysts	Fe (wt%)	Co (wt%)	Ni (wt%)	Mn (wt%)	Zn (wt%)
HELDH-SO_4^{2-}	6.48	6.31	7.01	4.68	5.66
HELDH-NO_3^-	7.42	6.64	8.87	5.05	6.35
HELDH-Cl^-	7.61	8.42	9.34	5.78	7.20

Table S2 Kinetic parameters of oxygen evolution reaction of electrode

Materials	C_{dl} (mF·cm^{-2})	ECSA (cm^2)	R_f (Ω·cm^2)
HELDH-SO_4^{2-}	1.26	31.5	31.5
HELDH-NO_3^-	1.02	25.5	25.5
HELDH-Cl^-	0.77	19.3	19.3

[a] ECSA = S×C_{dl} / C_s. S is the electrode surface area; C_s (the specific capacitance) is generally found to be in the range of 20 μF cm^{-2} – 60 μF cm^{-2} in 1.0 M KOH. In this work, we used the Cs = 40 μF cm^{-2}. The roughness factor R_f = C_{dl}/C_s.

Table S3. Estimated R_s, R_{ct}, and CPE based on Nyquist plots from EIS instrument

Catalyst	R_s (Ω)	R_{ct} (Ω)	CPE
HELDH-SO_4^{2-}	1.68	4.09	8.36×10^{-5}
HELDH-NO_3^-	1.79	7.38	1.51×10^{-4}
HELDH-Cl^-	1.85	9.83	5.72×10^{-4}

R_s refers to the resistance of the solution, CPE represents double-layer capacitance, R_{ct} denotes charge transfer impedance.

Table S4. Comparison of the OER performance of HELDH-SO_4^{2-} with recently reported catalysts in 1.0 M KOH.

Catalyst	η (mV @ 100 $mA\cdot cm^{-2}$)	Tafel (mV dec^{-1})	References
**HELDH-SO_4^{2-}**	**282**	**43.5**	**This work**
FeCoNiCuYP/C	316	64	Adv. Mater. 2024, 2410295.
(FeCoNiMnCu)O_x	365	85	Angew. Chem. 2023, 135, 202309854
NiCu-LDH	400	82	Adv. Funct. Mater. 2024, 34, 2309250.
D-(S)-Fe-Ni-OOH	350	60	Angew. Chem. 2024, 63, e202316762
$Ir_1/Ni_{1.6}Mn_{1.4}O_4$	330	75	Adv. Sci. 2022, 9, 2200529
F-$CoMoO_4$-x@GF	341	64	Appl. Catal. B 2022, 303, 120871.
CoNiRu-NT	335	67	Adv. Mater. 2022, 34, 2107488.
(CrMnFeCoNi)S_x	295	66	Adv. Energy Mater. 2021, 11, 2002887.
Ni-Mo-B HF	319	79	Adv. Funct. Mater. 2022, 32, 2107308
MIM@$Fe_{0.1}$-CoNi CH/NF	285	74	Chem. Eng. J, 2024, 491, 152023.
Fe-MoO_2/NF	340	75	Mater. Horiz., 2024, 11, 1199–1211.
NiCo@C-NiCoMoO	320	75	Nano-Micro Lett. 2021, 13, 77
S-(Ni,Fe)OOH	300	49	Energy Environ. Sci. 2020, 13, 3439.
a-NiCo/NC	310	49	Angew. Chem. Int. Ed. 2022, 134, 202207537
NiCo LDH	372	56	Adv. Funct. Mater. 2023, 33, 2304403
$Co(OH)(CO_3)_{0.5}$/NF	310	84.6	ACS Catal. 2023, 13, 13, 8821–8829
MoNiFe-27%	290	23	Nat. Commun. 2022, *13*, 2191.
Ru-S-NiFe LDH	279	81.65	Adv. Energy Mater. 2025, 15, 2500554
B_2-NiFe-(a-10)	290	100	Chem. Eng. J. 2024, 490, 151490
R-$NiFeO_xH_y$	272	44.7	Adv. Sci. 2023, 10, 2300717
L-TA-FeNi CP	280	45.4	Adv. Funct. Mater. 2023, 33, 2215051.

$(Ni_7Fe_3)OOH$-S	298	42.7	Chem. Eng. J. 2023, 454, 140030.
Co_9S_8-Ni_3S_2/NCF	302	51.5	Adv. Funct. Mater. 2025, 2419978
Co/CoMoN/NF	303	56	Adv. Sci. 2022, 9, 2105313.
Ni_2P-$NiFe_2O_4$	305	48.54	Appl. Catal. B Environ. 2023, 339, 123141.
CoNiRu-NT	335	67	Adv. Mater.2022, 34, 2107488.
F-$CoMoO_{4-x}$@GF	341	64.4	Appl. Catal. B Environ. 2022, 303, 120871.
$Ir_1/Ni_{1.6}Mn_{1.4}O_4$	330	75.0	Adv. Sci. 2022, 9, 2200529.
NiCo LDH	372	56.6	Adv. Funct. Mater. 2023, 33, 2304403.
D-(S)-Fe-Ni-OOH	350	60.0	Angew. Chem. Int. Ed. 2024, 63, e202316762.
NiCu-LDH	400	82.0	Adv. Funct. Mater. 2024, 34, 2309250.
$(R–COO)_x\sigma$-$Co_3O_{4−x}$	330	97.04	Nat. Chem. 2025. 16. 2483
h-$FeNi(OH)_x$-NiS@$Ni(OH)_2$/NF	295	72.4	Adv. Funct. Mater. 2024, 34, 2409849

* η_{100}, overpotential at 100 mA cm^{-2}. The overpotential and Tafel values are obtained from the literature.

Table S5 Performance comparison of AEM water electrolyzer from reported literature.

Cathode	Anode	Cell voltage	References
**Pt/C**	**HELDH-SO_4^{2-}**	**1.78**	**This work**
Er-CoP/NiCoP	Ce-Fe_xP/Ni_3P	1.78	Nano Lett. 2025, 25, 11, 4441
SM-CA-H	SM-CA-O	2.33	J. Mater. Chem. A 2024, 12, 29909.
NiFeMo-OV_R/NF	NiFeMo-OV_R/NF	1.86	Adv. Mater. 2024, 36, 2411134.
CoN/VN@NF	P-CoVO@NF	1.84	Adv. Mater. 2024, 36, 2408634.
PMoNiTm	NiFe-LDH	2.43	Chem. Eng. J. 2025, 508, 160921.
Raney Ni	(Ni,Fe)S_2@Ti_3C_2	1.81	Nat. Commun. 2025, 16, 1319.
NiSe/Fe-$Ni(OH)_2$	NiSe/Fe-$Ni(OH)_2$	1.79	China Chem. 2024, 67, 3468
Mn-NiFe@WO_x	Mn-NiFe@WO_x	1.83	Chem. Eng. J. 2024, 500, 157106.
NiMo alloy	PH-FCO	1.91	Small, 2025, p. 2505220.
PtRu/C (50 wt% Pt and 25 wt% Ru)	IrO_2	1.95	Nature Energy 2020, 5, 378-385
Pt black	$NiCoO_x$:Fe	2.45	ACS Catal., 2019, 9, 7-15
Pt/C	Fe_xNi_yOOH	1.94	ACS Catal., 2021, 11, 264-270
Co_3S_4	$Cu_{0·81}Co_{2·19}O_4$	2.20	Int. J. Hydrogen Energy,2020, 45, 36-45
NiMo	$Ni(OH)_2$-Fe	1.85	ACS Appl. Energy Mater., 2022, 5, 2221-2230
